# Notes from the Field Service School

**Published:** 1918-10

**Authors:** 


### THE SALVAGE OF SOLDIERS IN CONVALESCENT CAMPS

The salvage of soldiers who have been lightly wounded and the rapid return of those who are convalescent from disease, are problems of great importance to any Army. They are of particular interest to the medical officers of the American Expeditionary Forces at this time since American soldiers are fighting with our Allies on a large scale, thus making it necessary to care for many wounded men.

In this number of *War Medicine* is published an abstract of an address on this timely subject that was recently delivered by Colonel Bailey K. Ashford to the medical officers of the United States of the Sanitary Field Service School of the American Expeditionary Forces. Colonel Ashford has outlined a working plan for the salvage of convalescent sick and wounded soldiers that seems admirably adapted to the needs of our army. In prefacing his remarks on Convalescent Camps, Colonel Ashford said:

" There are at least four features in the work of the medical corps of an army today which may have recently placed medical officers in the position of being among the most active of the forces with which the enemy has to count; for by its medical corps an army *can* to a large extent delay and repair the ravages of war in men lost to the fighting line. In other words, the medical corps of an army has ceased to be a corps of doctors pure and simple, appendages of an army and non-combatant in every sense as far as war goes, who employ their profession to alleviate pain and repair the damaged individual. Today they constitute the most valuable of all salvage corps for men, the only corps by which damaged men *can* be salvaged. Naturally these officers are rewarded for the application of the medical sciences to the art of war by being deliberately bombed, gassed and shelled by an enemy who has not failed at all to realize the importance and value of their contribution. There are at least four outstanding factors in this new conception of the duties of a medical officer which make him willy-nilly an active participant in the winning of battles.

(1) The prevention of disease — by sanitary and by preventive medicine.

(2) The rapid collection and forwarding of the wounded and gassed, with intelligent first aid, such as the transportation of fractures in extension, the preventive treatment of shock, the immediate relief of the gassed, and the direct removal in the most comfortable and expeditious manner of all casualties to hospitals adequately provided to fulfill the requirements of modern military medicine and surgery.

(3) The prevention of infection of wounds by the surgical removal of the contaminating dead and dying tissue upon which organisms have heretofore been able to thrive; and

(4) The rapid return to duty, through convalescent camps, of men who heretofore have spent months in the zone of the interior, many never returning to the lines and being lost to the army. »

### Convalescent Camps.

« The whole idea of a convalescent camp or depot is: a point which the Medical Department operates to salvage men who would otherwise occupy a bed in a base hospital, or be sent back to their homes, or some watering place where the military spirit into which they have been inducted is more or less rapidly lost without any corresponding real benefit to their physique. The object is to get men away from base hospitals, who will need no further active surgical or medical treatment, but merely medical surveillance, and who especially need to be brought back into a military atmosphere, rather than be allowed to remain in a convalescent frame of mind. The two absolute essentials of a good convalescent camp are (1) that it should be a place which should combine all the pleasanter features of military life and its comforts, without supplying luxuries or permitting vices which tend to pamper the soldier and unfit him for his better return to the lines.

To show what can be done in this respect, Convalescent Camp No. 1, of the British, has returned to the Front direct 5,000 men in six months, and Convalescent Hospital No. 10 about 1,500 per month. In general the statements from the convalescent depots and camps seem to make it clear that the time spent in convalescence by men at base hospitals has by means of these camps been reduced from nine months to one month, in the 50 o/o of cases that are returned to duty at the Front.

These figures are approximate but were given me by their commanding officers. Lieutenant-General C. E. Burtchaell, Director Medical Services, British Expeditionary Forces in France, stated that out of 1,015 men at a convalescent camp, 756 were returned to duty cured as type " A " men, 127 remained as P. B., 123 were returned to base hospitals, and only *9* sent home to England.

In connection with the first essential of a good convalescent camp — that it should be run entirely by medical officers — it should be remembered that there are points at which damaged men are salvaged, and damaged men can not be salvaged save by the scientific medical officer whose aim and ambition is to put as many able bodied men back in the fighting line as soon as he can, and whose military sense is developed pari passu with his specialty. Such men can not be salvaged by mere doctors, nor by mere scientists, however well meaning they may be. This is the legitimate field of a well trained officer of the medical corps, who has really learned to apply his profession to the conditions of war. "

